# Low activity of complement in the cerebrospinal fluid of the patients with various prion diseases

**DOI:** 10.1186/s40249-016-0128-7

**Published:** 2016-05-03

**Authors:** Cao Chen, Yan Lv, Qi Shi, Wei Zhou, Kang Xiao, Jing Sun, Xiao-Dong Yang, Xiao-Ping Dong

**Affiliations:** State Key Laboratory for Infectious Disease Prevention and Control, National Institute for Viral Disease Control and Prevention, Chinese Center for Disease Control and Prevention, Chang-Bai Rd 155, Beijing, 102206 China; Collaborative Innovation Center for Diagnosis and Treatment of Infectious Diseases, Zhejiang University, Hangzhou, 310003 China; Chinese Academy of Sciences Key Laboratory of Pathogenic Microbiology and Immunology, Institute of Microbiology, Chinese Academy of Sciences, Beijing, 100101 China

**Keywords:** Prion diseases, CSF, Complement component, CH50, sCJD

## Abstract

**Background:**

The aim of this study was to analyze the state of activity and levels of complement in the cerebrospinal fluid (CSF) of patients with various prion diseases (PrDs).

**Findings:**

The proteomic data emphasized the levels of 20 known complement components found in the CSF of the sCJD panel that were lower than those found in the non-PrD panel. 50 % of the complement hemolytic activity (CH50) assays revealed significantly lower activity of complement in the CSF of the sCJD panel. The decreased levels of three key complement subunits, C3a/α, C4β, and C9 in the CSF of the sCJD panel were verified by Western blots. Furthermore, the CH50 values in the CSF of 136 sCJD, 39 gCJD, 22 FFI and 145 non-CJD patients were individually tested. Compared with the control of non-PrD, the CH50 value in the CSF specimens of various PrDs, especially in three subtypes of inherited PrDs, were significantly lower. Relationship analysis identified that the CH50 activity in the CSF was negatively associated with the protein 14–3–3 positive in the CSF.

**Conclusion:**

These results indicate a silent complement system in the CSF of PrD patients.

**Electronic supplementary material:**

The online version of this article (doi:10.1186/s40249-016-0128-7) contains supplementary material, which is available to authorized users.

## Multilingual abstracts

Please see Additional file 1 for translations of the abstract into the six official working languages of the United Nations.

## Background

Human prion diseases (PrDs) are a category of fatal neurodegenerative disorders including Creutzfeldt-Jakob disease (CJD), Gerstmann-Sträussler-Scheinker syndrome (GSS), fatal familial insomnia (FFI) and Kuru [1]. Surrogate biomarkers in CSF are screened for alterations, which not only identify the diagnostic markers for CJD, but also provide insight for understanding the pathogenesis. So far, only the immunoblot for the 14–3–3 protein in CSF is included in the diagnostic criteria for sporadic CJD (sCJD) [2].

The complement system, which consists of at least 30 kinds of soluble and membrane-bound proteins, is an immune-defense mechanism with a wide range of effects that take place in the central nervous system (CNS). Evidence suggests that the activation of complement components occurs in patients with various neurodegenerative disorders [3–6], and that certain complement proteins are deregulated during prion infection [5, 7–12]. Our previous proteomic study has revealed that some complement components were lower in the CSF of sCJD patients [13]. However, the exact relationship has yet to be proven.

## Methods

This study was approved by the Ethical Committee of the National Institute for Viral Disease Control and Prevention, China CDC, under protocol 2009ZX10004–101.

To verify the changes of complement in the CSF of sCJD patients according to the proteomic code, we reviewed 37 CSF samples from probable sCJD patients who tested positive for 14–3–3 protein in their CSF and typical periodical sharp-wave complexes (PSWC) in their EEG. We also analyzed 36 CSF samples from non-CJD patients, who were CSF 14–3–3 negative and had normal levels of EEG. These samples were taken from the CSF bank in the China CJD Surveillance Center (Additional file 2: Table S1). 50 μl CSF samples were taken from each group and labeled sCJD or non-CJD, respectively. Furthermore, the CSF samples were further subdivided as such: 136 sCJD, 39 genetic CJD (gCJD), 22 FFI and 145 non-CJD (Table 1). This allowed us to screen the complement activity individually. The possible clinical diagnoses of those 145 non-CJD cases were summarized in Additional file 3: Table S2.

**Table 1:** The relevant characteristics of the patients with non-CJD and sCJD, gCJD and FFI

Clinical features	non-CJD	sCJD	gCJD		FFI
			T188K	E200K	
Number (n)	145	136	25	14	22
Gender (M/F)	71/74	72/64	16/9	7/7	9/13
Median age at onset (range) (y)	61 (45–89)	65 (47–81)	50 (40–85)	56 (44–68)	47.5 (19–63) ^a^
Age at onset <50 years (%)	7(4.8)	3 (2.2)	2 (8)	4 (28.6)	12 (54.5)
Age at onset 50–70 years (%)	114 (78.6)	96 (70.6)	17 (68)	10 (71.4)	10 (45.5)
Age at onset >70 years (%)	24 (16.6)	37 (27.2)	6 (24)	0 (0)	0 (0)
Patients with *PRNP* gene sequenced (%)	145 (100)	136 (100)	25 (100)	14 (100)	22 (100)
Codon 129 genotype					
Met-Met/Total (%)	143/145 (98.6)	133/136 (97.8)	13/25 (100)	14/14 (100)	22/22 (100)
Met-Val/Total (%)	2/145 (1.4)	3/136 (2.2)	0/25 (0)	0/14 (0)	0/22 (0)
Val-Val/Total (%)	0/145 (0)	0/136 (0)	0/25 (0)	0/14 (0)	0/22 (0)
EEG Typical/Total (%)	9/145 (6.2)	92/136 (67.6) ^b^	3/25 (12)	8/14 (57.1) ^b^	0/22 (0)
14–3–3 Positive/Total (%)	29/145 (20)	99/136 (72.8) ^b^	18/25 (72) ^b^	14/14 (100) ^b^	6/22 (27.2)
Progressive dementia/Total (%)	121/145 (83.4)	128/136 (94.1) ^b^	20/25 (80)	13/14 (92.9)	17/22 (77.3)
Myoclonus (%)	33/145 (22.8)	106/136 (77.9) ^b^	15/25 (60) ^b^	10/14 (71.4) ^b^	12/22 (54.5) ^b^
Visual or cerebellar disturbance (%)	22/145 (15.2)	88/136 (64.7) ^b^	18/25 (72) ^b^	11/14 (78.6) ^b^	11/22 (50) ^b^
Pyramidal or extramidal dysfunction (%)	70/145 (48.3)	104/136 (76.5) ^b^	19/25 (76)^c^	12/14 (85.7) ^c^	13/22 (59.1)
Akinetic mutism (%)	24/145 (16.6)	66/136 (48.5) ^b^	10/25 (40)^c^	8/14 (57.1) ^b^	3/22 (13.6)

^a^ compared with non-PrD controls by Mann–Whitney *U* test and the superscript of “a” means *P* < 0.01

^b/c^ compared with non-PrD controls through Chi-Square test and the superscript of “b” represents p < 0.01 and that of “c” indicates *P <* 0.05

All enrolled CSF samples were obtained by standard clinical procedures and were free of blood contamination. Routine CSF biochemistry assays of those specimens, including cell count, glucose, and total protein were all within the normal ranges.

The detection of the 50 % of complement hemolytic activity (CH50, U/ml) in CSF (50 μl of each tested sample) was derived from the commercial double-antibody sandwich enzyme-linked immunosorbent assays (ELISAs, YAD, China). Western blots for the complement components C3a/α, C4β and C9 in the panels of the pooled CSF (20 μl of each tested sample) were conducted using anti-C3a/α mAbs (sc-47688), anti-C4β pAbs (sc-25816) and anti-C9 mAbs (sc-390000) (Santa Cruz biotechnology Inc). The detailed procedures of CH50 ELISA and Western blot are described elsewhere [5]. The data was processed with GraphPad Prism 5 and SAS 9.2 statistics software.

## Findings

Based on the raw data of our previous study [13], we identified 20 complement-related components that were differentially expressed in the sCJD group (Table 2). All those identified complement components in the CSF of the sCJD group were down-regulated compared with those of non-CJD group, among which were 16 proteins that showed significant statistical difference (*P* < 0.01). 5 out of 20 complement components were statistically different at the 95 % confidence interval (gray box in Table 2) by the Peptide Prophet algorithm [14], including C4-B, C7, C2, C9 and factor B.

**Table 2:**
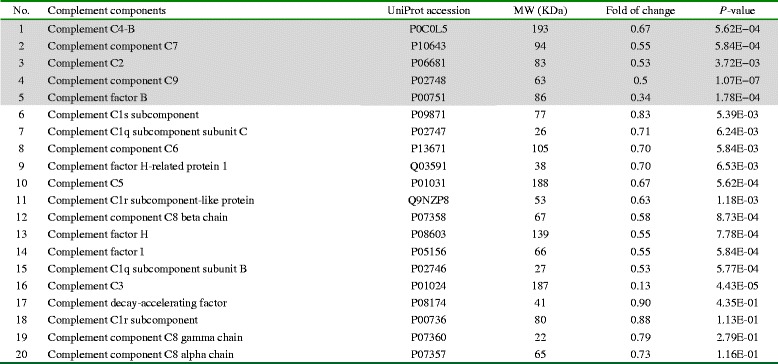
Comparison of the complement proteins in CSF between the groups of probable sCJD and non-CJD in proteomic assay [11]

**Fig. 1:**
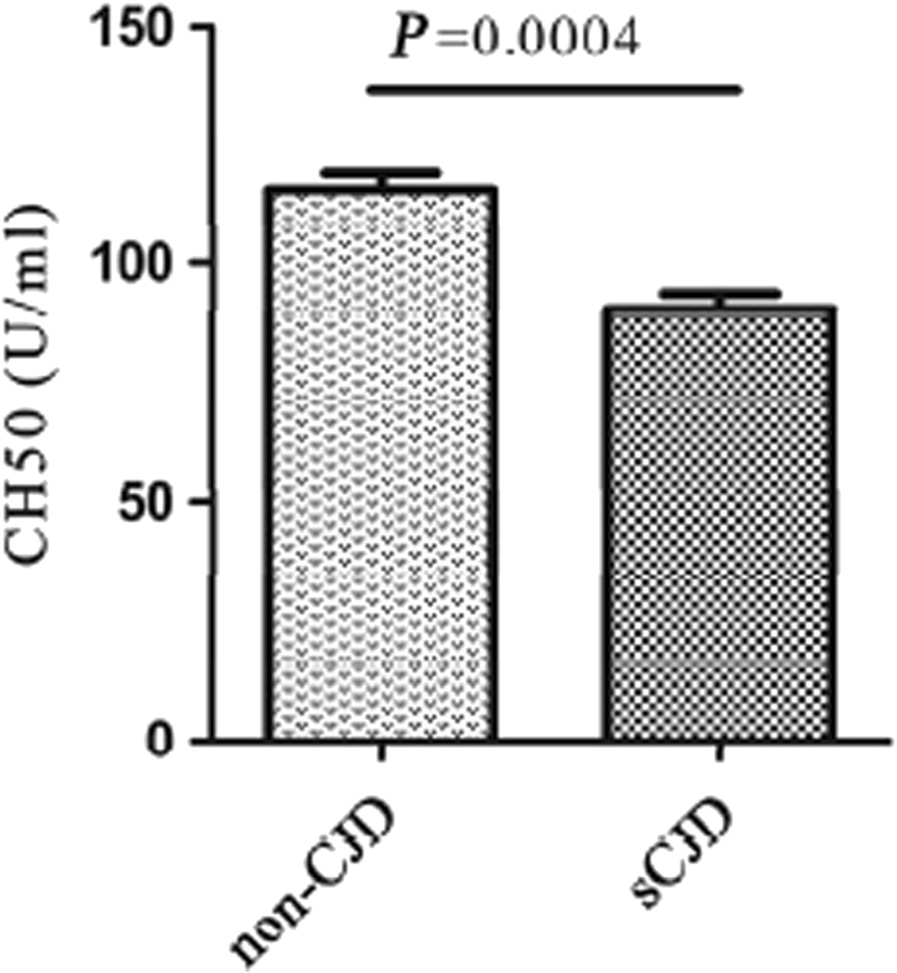
Indirect ELISAs which indicate 50 % of complement hemolytic activity (CH50, U/ml) in the pooled CSF samples of sCJD and non-PrDs subjects. The vertical axis represents the unit of CH50. The results are calculated with data from three independent assays and presented as mean ± SEM

**Fig. 2:**
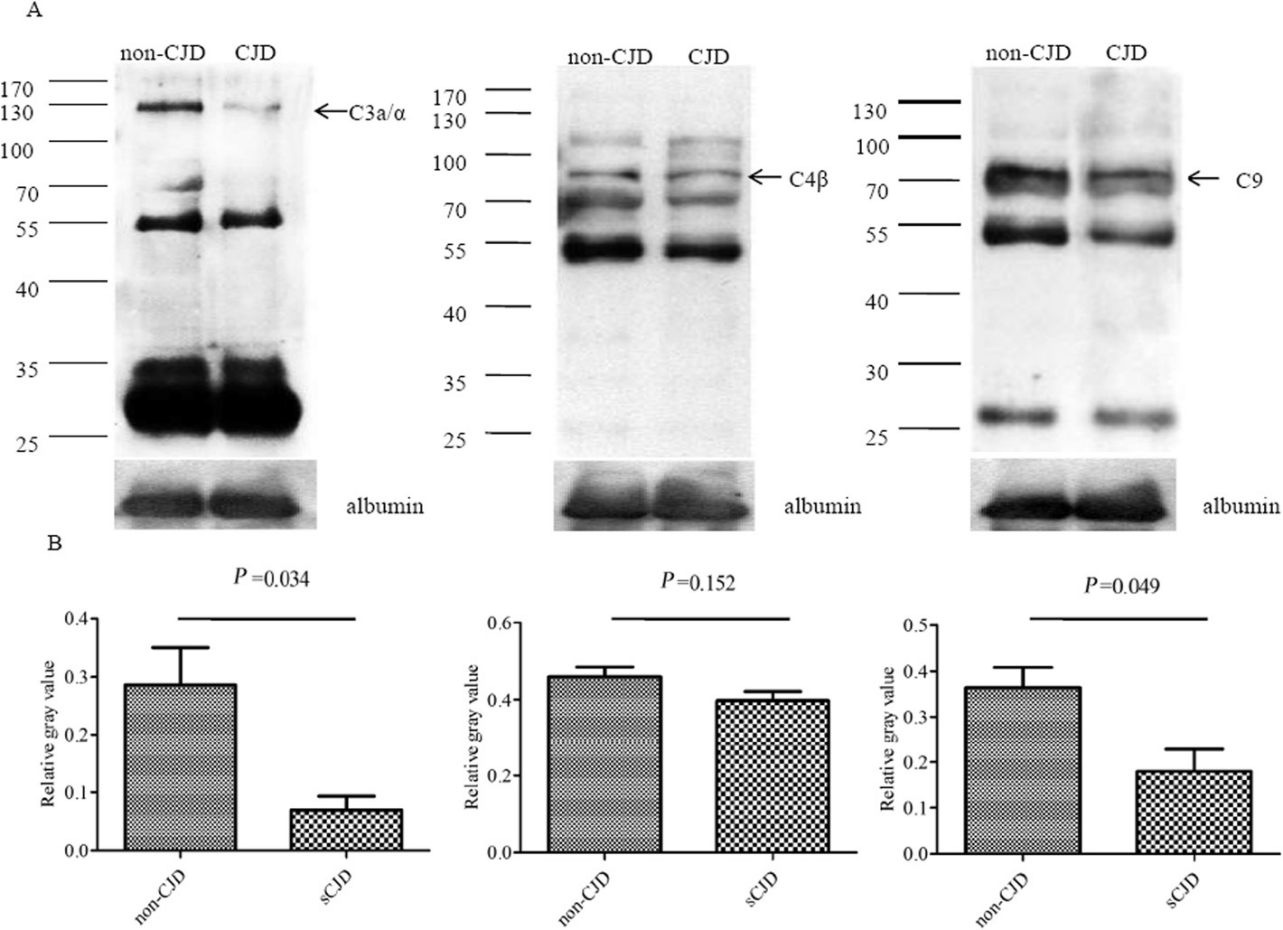
Evaluation of the levels of three key complement subunits in the pooled CSF of sCJD and non-CJD subjects. **a** Western blots of the CSF panels of sCJD and non-CJD subjects using C3a/α, C4β, and C9-specific antibodies. Albumin in CSF was used as an internal control. **b** Quantitative analyses of the averaged gray values normalized with the albumin of individual panels are shown below. The average gray values are calculated with the data of three independent assays and presented as mean ± SD

To obtain more evidence for the alteration of complement activity and complement components in the CSF of sCJD patients, CH50 in the CSF panels of sCJD and non-CJD (protein concentration: 283 ± 15 mg/l for the sCJD panel and 315 ± 23 mg/l for the non-CJD panel) were analyzed using a commercial indirect ELISA kit. Compared with the panel of non-CJD, CH50 in the sCJD panel was significantly lower (Fig. 1, *P* < 0.001). Furthermore, the decreased levels of three key subunits of complement C3a/α, C4β, and C9 in complement cascades in the CSF panel of sCJD were observed by Western blots (Fig. 2).

**Fig. 3:**
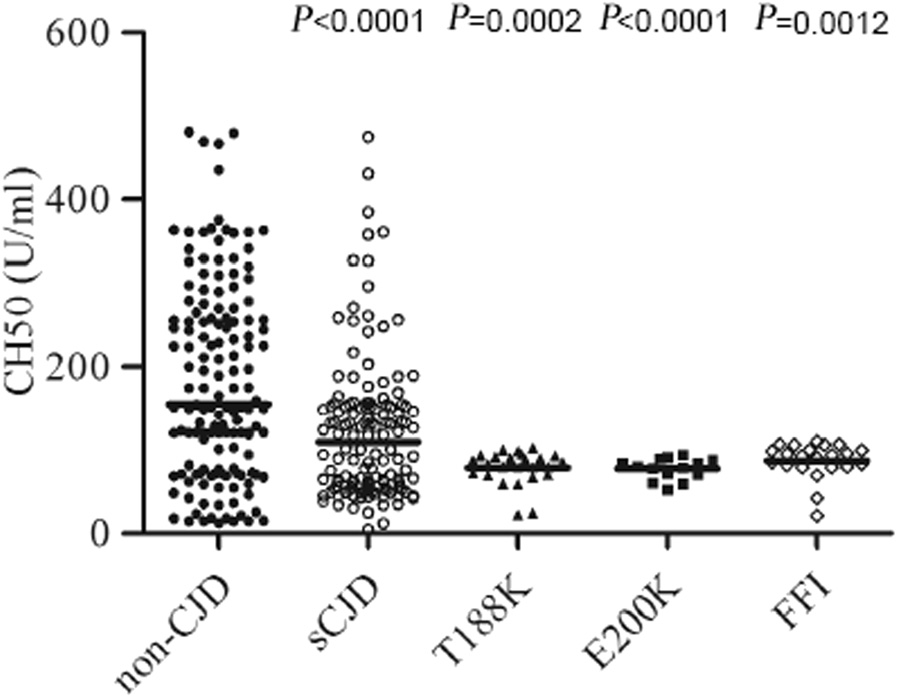
Detection of individual CSF levels of the total complement activity among a sampling of human prion diseases using indirect ELISAs which indicate 50 % of complement hemolytic activity. The solid line within each group represents the median of CH50 values. The P values between the groups of non-CJD subjects and individual prion diseases are labeled above

To evaluate CH50 activity in the CSF of PrD patients, CSF samples from 197 cases of various PrDs and 145 CSF samples from non-PrDs were given CH50 assays separately. As shown in Fig. 3, the median CSF CH50 values in the group of sCJD, T188K-gCJD, E200K-gCJD, and FFI were significantly lower than that of non-CJDs. Further analysis showed that the CH50 values in 49.3 % (67/136) of sCJDs, 92.0 % (23/25) of T188K gCJDs, 100 % (14/14) E200K gCJDs, and 77.3 % (17/22) of FFI were below 100 U/ml, whereas only 28.3 % (41/145) of non-CJD were in this zone (Table 3). This data indicates a profile of lower CH50 values in the CSF of PrD patients. Unlike the wide distribution of CH50 in samples taken from sCJD and non-CJD groups, the distribution of CSF CH50 values in T188K-, E200K-gCJD, and FFI were much narrower. Additionally, the CSF CH50 values do not correspond with the intervals from the aforementioned diseases onset to sampling in all tested groups (Additional file 4: Table S3). Nor do they influence the survival time of the 31 sCJD patients (Additional file 5: Figure S1).

**Table 3:** The numbers of CSF samples among different human prion diseases according to various CH50 value ranges

Groups	CH50 (U/ml) Median (min, max)	*P*-value^a^	CH50 (U/ml) % (case No./group No.)			
			<100	100–200	200–300	>300
non-CJD	154.066 (12.407–480.312)	-	28.3 % (41/145)	29.7 % (43/145)	24.1 % (35/145)	17.9 % (26/145)
sCJD	108.55 (4.56–474.40)	<0.0001	49.3 % (67/136)	38.2 % (52/136)	7.4 % (10/136)	5.1 % (7/136)
T188K	88.432 (22.39–109.237)	0.0002	92 % (23/25)	8 % (2/25)	0	0
E200K	76.577 (52.03–94.03)	<0.0001	100 % (14/14)	0	0	0
FFI	92.905 (21.43–109.70)	0.0012	77.3 % (17/22)	22.7 % (5/22)	0	0

^a^ Mann–Whitney *U* test

**Fig. 4:**
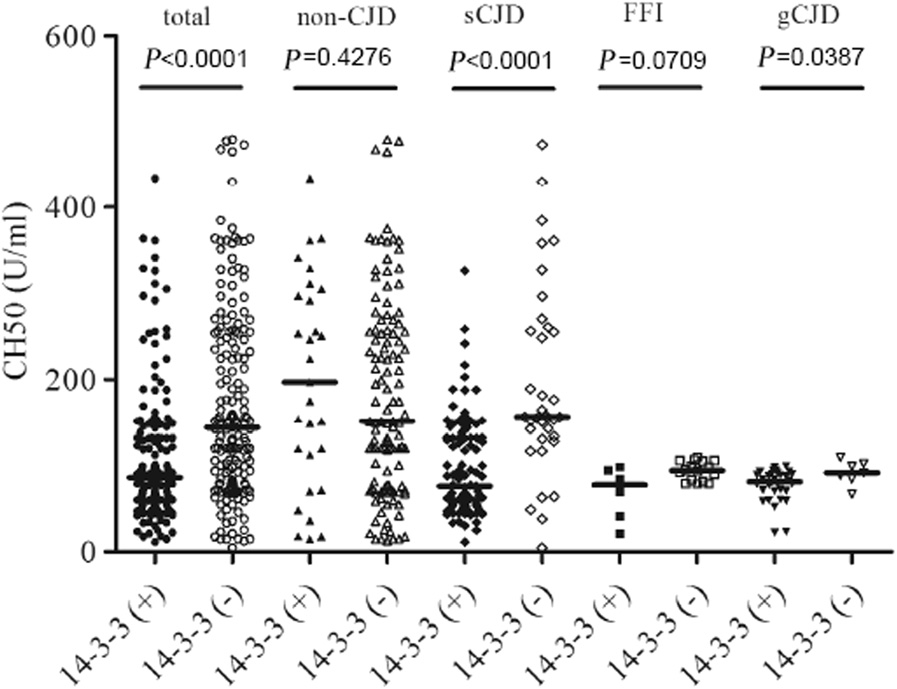
The distributions of CH50 activity in the CSF among various PrD subjects based on whether they are 14-3-3 positive or negative. The solid line within each group represents the median of CH50 values. *P* values between the groups of 14–3–3 positive and negative are indicated above

To address the potential factor(s) correlated with the CSF CH50 activity, some clinical and laboratory parameters from the enrolled patients were statistically analyzed. Univariate analysis illustrated a significant correlation between 14–3–3 and CH50 (Table 4, *P* = 0.003). Further multivariate logistic regression proposed that only the presence of 14–3–3 in CSF showed the notable correlation with CSF CH50 activity (*P* = 0.004). To further evaluate the relationship between CH50 activity and 14–3–3 positive CSF, all enrolled cases were grouped depending if they were 14–3–3 positive or negative. As shown in Fig. 4, in the context of 342 tested samples, the CSF CH50 values of the cases that were 14–3–3 positive (median: 86.03 U/ml) were clearly lower than those that were 14–3–3 negative (median: 143.6 U/ml) (*P* < 0.0001). Subsequent assays based on various diagnoses illustrated lower CSF CH50 activity in the sCJD cases that were 14–3–3 positive (*P* < 0.0001). Non-CJD cases that tested 14–3–3 positive, in contrast, tested relatively higher without statistical difference (*P* = 0.545) (Fig. 4). Lower CH50 values were also detected in the cases of genetic PrDs that were 14–3–3 positive, but those values were only significant in the gCJD group (*P* = 0.0387). This demonstrates a negative correlation of lower CSF levels and CH50 activity with 14–3–3 positive PrD patients.

**Table 4:** Multivarivate logistic regression of the levels of CSF CH50 and relational influence factors

	Factors	*OR* 95 % (*CI*)	*P*-value
Univariate analysis	Gender		
	Male	0.793 (0.456, 1.376)	0.409
	Female	Reference	-
	Age of onset		
	<50	1.260 (0.343, 4.623)	0.728
	50–70	1.503 (0.713, 3.169)	0.285
	>70	Reference	
	Codon 129 genotype		
	Met-Met	1.114 (0.107, 11.648)	0.928
	Met-Val	Reference	
	EEG		
	n.d.	0.231 (0.127, 1.003)	0.272
	Positive	0.684 (0.348, 1.346)	
	Negative	Reference	
	14–3–3		0.003
	Positive	0.372 (0.195, 0.707)	
	Negative	Reference	
	Progressive dementia		0.814
	Yes	1.104 (0.485, 2.510)	
	No	Reference	
	Myoclonus		0.703
	Yes	1.137 (0.588, 2.199)	
	No	Reference	
	Visual or cerebellar disturbance		0.165
	Yes	0.639 (0.340, 1.202)	
	No	Reference	
	Pyramidal or extramidal dysfunction		0.090
	Yes	0.602 (0.335, 1.082)	
	No	Reference	
	Akinetic mutism		0.314
	Yes	0.692 (0.337, 1.419)	
	No	Reference	
multivarivate logistic regression	14–3–3		0.004
	Positive	0.362 (0.182, 0.722)	
	Negative	Reference	

n.d. represents not detected

This study has been the first time that lower CSF complements in PrD patients have been observed. The changes in complement components in CSF have either decreased [15] or increased [4]. However, the CSF samples in those studies are not from a healthy control group, but from patients with various neurological disorders. Activation of the complement system is frequently detected in the brains of PrD patients. The reason for this remains unclear, in addition to the relatively silent complement. It might be assumed that the recruitment of the large amount of complement components in the brain [3, 5], such as forming C1q-PrP^Sc^ complex [16, 17] and membrane attack complex (MAC) [5], might lead to smaller amounts of complement penetrating CSF.

Unlike the wide-range distribution of CSF CH50 values in sCJD, the CH50 values in the three subtypes of genetic PrDs are low with a narrow range. It indicates a silent state of the innate immune system in the central nervous system during the pathogenesis of genetic PrDs. In accordance with our previous study, microglia and some cytokines are silent in the post-mortem brains of FFI and G114V gCJD cases, while some are obviously activated in other types of sCJD cases [18]. Recent iTRAQ-based proteomic study of the brain tissues of sCJD, FFI, and G114V gCJD show multiple pathways involving immunity and infection in the brains of sCJD subjects, except for hereditary cases [19]. Additionally, the diversity of clinical and pathological characteristics in sCJD may also be contributing factors to the variation in CH50 present within CSF.

14–3–3 positive CSF samples are the unique factor correlated with CH50 values found in the CSF of sCJD patients. The exact molecular association between these two factors is unknown. Contrary to the well-known diagnostic evidence for sCJD, the correlation of 14–3–3 CSF samples with brain pathology in CJD are rarely reported, and draw controversial conclusions [20–22]. 14–3–3 samples are also associated with the alterations of other proteins in the CSF of sCJD patients. Our recent publication illustrates that higher levels of tau isoforms containing Exon-2 and Exon-10 segments in sCJD patients with 14–3–3 positive CSF [23]. The NSE level in CSF of sCJD patients is also associated with 14–3–3 positive readings [24]. In that case, one might assume that 14–3–3 positive CSF samples of sCJD patients correlate with the alterations of CH50 values and other biomarkers in CSF, thus representing a (sCJD) disease-associated feature. Nevertheless, the exact molecular mechanism of 14–3–3 positive and lower CH50 levels in the CSF of sCJD patients deserves further study.

## Conclusions

The complement activities in the CSF of patients with prion diseases (including sCJD, gCJD, and FFI), are lower than their non-CJD counterparts are. Additionally, subjects whose CSF samples tested 14–3–3 positive exhibit lower complement activity in sCJD patients.

## Additional files

Additional file 1: Multilingual abstracts in the sex official working languages of the United Nations. (PDF 237 kb) Additional file 2: Table S1. The relevant characteristics of CSF samples between the group of probable sCJD and non-CJD. (DOCX 24 kb) Additional file 3: Table S2. The clinical diagnoses of 145 non-CJD cases. (DOCX 23 kb) Additional file 4: Table S3. Analysis of CH50 values in CSF from various prion diseases according to the intervals from disease onset to sampling. (DOCX 20 kb) Additional file 5: Figure S1. Description of CH50 values present within CSF from sampling until respective subject’s death, derived from 31 sCJD cases. X-axis represents the interval time and Y-axis represents the CH50 values present within the CSF. P values among the groups are indicated above. (TIF 46 kb)
