# Supplementary material for: Low activity of complement in the cerebrospinal fluid of the patients with various prion diseases
Source: Infect Dis Poverty. 2016 May 3;5:35. doi: 10.1186/s40249-016-0128-7 (PMC4853859; doi:10.1186/s40249-016-0128-7)
Supplement: Additional file 1: — Multilingual abstracts in the sex official working languages of the United Nations. (PDF 237 kb) [file 40249_2016_128_MOESM1_ESM.pdf]

Translation of the abstract into the six official working languages of the United Nations

الفعالية المنخفضة للمتممة في السائل الدماغي الشوكي للمرضى المصابين بأمراض البريون المختلفة

تشاو تشين، يان ليو، كي شي، ووي زهو، كانغ زياو، جينغ سن، زياو-دونغ-يانغ، زياة بينغ دونغ

موجز

الخلفية: كان الهدف من هذه الدراسة هو تحليل درجة الفعالية ومعدلات المتممة في السائل الدماغي الشوكي (CSF) للمرضى المصابين لأحراض البريون المختلفة (PrDs).

الموجودات: أكدت البيانات البروتيومية على معدل العشرين متممة المعروفة والتي توجد في السائل الدماغي الشوكي لمرضى كروتزفيلد-جاكوب الفرادي والتي كانت أقل من تلك الموجودة في مجموعة الأمراض غير البريونية. كشفت مقايسات 50% من فعالية المتممات الحالة للدم **(CH50)** عن فعالية أقل بوضوح من المتممات في السائل الدماغي الشوكي لدى مرضى كروتزفيلد جاكوب. وقد جرى التحقق من المعدلات المتناقصة لثلاث وحدات فرعية للمتممات الرئيسية، C3a/α، C4β، وC9 في السائل الدماغي الشوكي لمرضى كروتزفيلد-جاكوب الفرادي، بواسطة لطاخات وسترن. إلى جانب هذا، جرى اختبار فردي لقيم **CH50** في السائل الدماغي الشوكي لـ 136 مصاب بمرض كروتزفيلد-جاكوب الفرادي، 39 مصاب بمرض كروتزفيلد-جاكوب الوراثي، 22 حالة أرق عائلي مميت و145 خالة غير مصابة بمرض كروتزفيلد جاكوب. عند مقارنتها بالأرقام المرجعية لغير المصابين بمرض بريوني، فإن **CH50** في عينات السائل الدماغي الشوكي المأخوذة من أمراض بريونية متنوعة، وخاصة في ثلاث أنماط فرعية من أمراض البريون الموروثة، كانت أقل بشكل ملحوظ. وقد حدد تحليل العلاقة أن فعالية **CH50** في السائل الدمافي الشوكي كانت مرتبطة سلبيا بالبروتين **14-3-3** الإيجابي في السائل الدماغي الشوكي.

الاستنتاج: تشير هذه النتائج إلى وجود نظام متمم صامت في السائل الدماغي الشوكي للمرضى الذين يشكون من أمراض بريونية.

Translated from English version into Arabic by Lina SM, through

TRANSLATORS WITHOUT BORDERS

不同类型朊病毒病患者脑脊液呈现较低的补体活性

陈操、吕燕、石琦、周伟、肖康、孙静、杨晓东、董小平

摘要：

引言： 本研究旨在分析不同类型朊病毒感染患者脑脊液中补体的活性和含量。

结果： 蛋白质组学研究发现散发型克雅氏病（朊病毒病中的一种重要类型）患者脑脊液中有 20 种已知的补体成分含量明显低于非朊病毒病患者。通过 50%补体溶血活性检测（CH50）发现在散发型克雅氏病患者脑脊液中补体总活性明显偏低，随后我们通过 Western blot 检测了脑脊液中三种关键补体成份的含量（C3a/α、C4β 和 C9），发现散发型克雅氏病患者均低于非克雅氏病患者。进一步，我们对 136 例散发型克雅氏病、39 例遗传型克雅氏病、22 例家族致死性失眠症及 145 例非朊病毒病患者脑脊液中 CH50 值分别进行了检测。与非克雅氏病患者相比，不同朊病毒病患者脑脊液中 CH50 均明显偏低，尤其是在遗传型朊病毒病中。相关性分析显示这些患者脑脊液补体活性与脑脊液 14-3-3 蛋白阳性呈负相关。

结论： 本研究结果提示朊病毒病患者脑脊液中补体系统呈现“静默”的状态。

Translated from English version into Chinese by Cao Chen

Faible activité du complément dans le liquide céphalo-rachidien de patients atteints de différentes maladies à prions

Cao Chen, Yan Lyu, Qi Shi, Wei Zhou, Kang Xiao, Jing Sun, Xiao-Dong Yang, Xiao-Ping Dong

Résumé

Contexte: la présente étude a pour objectif d’analyser l’état d’activité et les concentrations du complément dans le liquide céphalo-rachidien (LCR) de patients atteints de différentes maladies à prions (MaPr).

Observations: les données protéomiques ont souligné les concentrations de 20 composants du complément connus et identifiés dans le LCR du groupe à MCJ sporadique qui étaient inférieures à celles mesurées dans le groupe non MaPr. 50 % des essais de mesure de l’activité hémolytique complémentaire (CH50) ont révélé une activité nettement inférieure du complément dans le LCR du groupe à MCJ sporadique. La baisse des concentrations des trois principales sous-unités du complément que sont le C3a/α, le C4β, et le C9 dans le LCR du groupe à MCJ sporadique a été vérifiée par des essais « western blot ». De plus, les valeurs du CH50 dans le LCR de 136 patients atteints d’une MCJ sporadique, de 39 patients atteints d’une MCJ génétique, de 22 patients atteints d’une IFF et de 145 patients non atteints par une MCJ ont été analysées de manière individuelle. Par rapport au contrôle des patients non atteints par une MaPr, la valeur du CH50 dans les échantillons de LCR de différents patients atteints d’une MaPr, notamment dans les trois sous-types de MaPr héréditaire, était significativement plus faible. Une analyse des relations a permis de déterminer que l’activité du CH50 dans le LCR était associée de manière négative à la protéine 14-3-3 positive dans le LCR.

Conclusion: ces résultats indiquent la présence d’un système complémentaire silencieux dans le LCR de patients atteints d’une MaPr.

Translated from English version into French by Eric Ragu, through

TRANSLATORS
WITHOUT BORDERS

Низкая активность комплемента в спинномозговой жидкости пациентов с различными прионными заболеваниями

Цао Чжэнь, Янь Лу, Ци Ши, Вэй Чжоу, Ган Сяо, Цзин Сунь, Сяо-Дун Ян, Сяо-Пин Дун

Краткое изложение

История вопроса: Цель данного исследования – проанализировать состояние активности и уровни комплемента в спинномозговой жидкости (СМЖ) пациентов с различными прионными заболеваниями (ПЗ).

Результаты исследования: Протеомные данные подчеркнули уровни 20 известных компонентов комплемента, найденных в СМЖ панели пациентов со спорадической формой болезни Крейтцфельдта-Якоба (sCJD), которые были ниже, чем уровни панели пациентов без ПЗ. Анализы 50% гемолитической активности комплемента (CH50) показали значительно более низкую активность комплемента в СМЖ панели сГЭКРС. Пониженные уровни трех основных субъединиц комплемента, C3a/α,

C4β и C9 в СМЖ панели sCJD были подтверждены вестерн-блоттингом. Более того, были индивидуально протестированы значения CH50 в СМЖ 136 пациентов с sCJD, 39 пациентов с gCJD, 22 пациентов с фатальной семейной бессонницей (FFI) и 145 пациентов без CJD. В сравнении с контрольной группой пациентов без ПЗ, значение CH50 в образцах СМЖ пациентов с различными ПЗ, особенно трех видов унаследованных ПЗ, было значительно ниже. Анализ зависимости выявил, что активность CH50 в СМЖ была отрицательно связана с повышенными уровнями белков 14-3-3 в СМЖ.
Заключение: Эти результаты указывают на пассивную систему комплемента в СМЖ пациентов с ПЗ.

Translated from English version into Russian by Elena McDonnell, through

TRANSLATORS
WITHOUT BORDERS

Baja actividad del complemento en el líquido cefalorraquídeo de pacientes con diversas enfermedades priónicas

Cao Chen, Yan Lyu, Qi Shi, Wei Zhou, Kang Xiao, Jing Sun, Xiao-Dong Yang, Xiao-Ping Dong

Resumen
Antecedentes: El objeto del presente estudio fue analizar el estado de actividad y los niveles de complemento en el líquido cefalorraquídeo (LCR) en pacientes con diversas enfermedades priónicas.
Hallazgos: Los datos proteómicos pusieron de relieve los niveles de 20 componentes conocidos del complemento en el LCR del panel de ECJ esporádica, los que fueron más bajos que aquellos encontrados en los paneles sin enfermedad priónica. 50% de los ensayos de actividad hemolítica del complemento (CH50) revelaron una actividad significativamente reducida del complemento en el LCR del panel de ECJ esporádica. Los niveles disminuidos de tres subunidades claves del complemento, C3a/α, C4β, y C9 en el LCR del panel de ECJ esporádica fueron verificados mediante prueba de Western blot. Es más, se evaluaron de forma individual los valores del CH50 en el LCR de 136 pacientes con ECJ esporádica, 39 pacientes con ECJ genética, 22 pacientes con IFF y 145 pacientes sin ECJ. Comparados con el control sin enfermedades priónicas, el valor del CH50 en los especímenes de LCR de varias enfermedades priónicas, en particular en tres subtipos de enfermedades priónicas hereditarias, fue significativamente más bajo. El análisis de relación identificó que la actividad del CH50 en el LCR se veía negativamente asociada a positividad de la proteína 14-3-3 en el LCR.
Conclusión: Estos resultados indican un sistema de complemento silencioso en el LCR de pacientes con enfermedades priónicas.

Translated from English version into Spanish by Maria Alejandra Aguada, through

TRANSLATORS
WITHOUT BORDERS
