# Supplementary material for: Low activity of complement in the cerebrospinal fluid of the patients with various prion diseases
Source: Infect Dis Poverty. 2016 May 3;5:35. doi: 10.1186/s40249-016-0128-7 (PMC4853859; doi:10.1186/s40249-016-0128-7)
Supplement: Additional file 2: Table S1. — The relevant characteristics of CSF samples between the group of probable sCJD and non-CJD. (DOCX 24 kb) [file 40249_2016_128_MOESM2_ESM.docx]

Supplemental Table 1. The relevant characteristics of CSF samples between the group of probable sCJD and non-CJD

| Clinical features | Probable sCJD | Non-CJD | *p*-value |
| --- | --- | --- | --- |
| Number (n) | 37 | 36 |  |
| Gender ratio (M:F) | 19:18 | 13:5 | 0.067^b^ |
| Median age at onset (range) (y) | 63 (46-86) | 58 (44-77) | 0.118^a^ |
| Age at onset <50 years (%) | 2 (5.4) | 2 (5.6) | 1.000^b^ |
| Age at onset 50-70 years (%) | 26 (70.3) | 27 (75) | 0.651^b^ |
| Age at onset >70 years (%) | 9 (24.3) | 7 (19.4) | 0.614^b^ |
| Patients with *PRNP* gene sequenced (%) | 37/37 (100) | 36/36 (100) | - |
| Codon 129 genotype |  |  |  |
| Met-Met/total (%) | 37/37 (100) | 36/36 (100) | - |
| Met-Val/total (%) | 0/37 (0) | 0/36 (0) | - |
| Val-Val/total (%) | 0/37 (0) | 0/36 (0) | - |
| EEG Typical/ total (%) | 37/37 (100) | 0/36 (0) | - |
| 14-3-3 Positive/total (%) | 37/37 (100) | 0/36 (0) | - |
| Progressive dementia/Total (%) | 37/37 (100) | 14/36 (38.9) | p<0.01^b^ |
| Myoclonus (%) | 31/37 (83.8) | 2/36 (5.6) | p<0.01^b^ |
| Visual or cerebellar disturbance (%) | 25/37 (67.6) | 4/36 (11.1) | p<0.01^b^ |
| Pyramidal or extramidal disfunction (%) | 30/37 (81.1) | 7/36 (19.4) | p<0.01^b^ |
| Akinetic mutism (%) | 14/37 (37.8) | 1/36 (2.8) | p<0.01^b^ |

^a^Mann-Whitney *U*-test.

^b^Chi-Square test.
