# Supplementary material for: Low activity of complement in the cerebrospinal fluid of the patients with various prion diseases
Source: Infect Dis Poverty. 2016 May 3;5:35. doi: 10.1186/s40249-016-0128-7 (PMC4853859; doi:10.1186/s40249-016-0128-7)
Supplement: Additional file 3: Table S2. — The clinical diagnoses of 145 non-CJD cases. (DOCX 23 kb) [file 40249_2016_128_MOESM3_ESM.docx]

Supplemental Table 2. The clinical diagnoses of 145 non-CJD cases

| Order No. | Clinical diagnosis | Case No. |
| --- | --- | --- |
| 1 | Cerebral infarction | 41 |
| 2 | Senile dementia | 35 |
| 3 | Viral encephalitis | 20 |
| 4 | Epilepsy | 7 |
| 5 | Pakinson disease | 5 |
| 6 | Vascular dementia | 5 |
| 7 | NMDA receptor encephalitis | 3 |
| 8 | Limbic encephalitis | 3 |
| 9 | Delayed encephalopathy after carbon monoxide poisoning | 3 |
| 10 | Subacute Combined Degeneration | 3 |
| 11 | Paraneoplastic encephalomyelitis | 2 |
| 12 | Alzheimer’s disease | 2 |
| 13 | Cerebral cancer | 2 |
| 14 | Neurosyphilis encephalopathy | 2 |
| 15 | Hashimoto’s encephalopathy | 1 |
| 16 | Hepatic encephalopathy | 1 |
| 17 | Acute myelitis | 1 |
| 18 | Brain metastasis from lung cancer | 1 |
| 19 | Progressive supranuclear palsy | 1 |
| 20 | Hyponatremia | 1 |
| 21 | Diabetic complication | 1 |
| 22 | Mitochondrial encephalomyopathy | 1 |
| 23 | Toxicencephalitis | 1 |
| 24 | Cytomegalovirus infection | 1 |
| 25 | Drug allergy | 1 |
| 26 | Encephalomyelopath | 1 |
| Total | | 145 |
