# Supplementary material for: Low activity of complement in the cerebrospinal fluid of the patients with various prion diseases
Source: Infect Dis Poverty. 2016 May 3;5:35. doi: 10.1186/s40249-016-0128-7 (PMC4853859; doi:10.1186/s40249-016-0128-7)
Supplement: Additional file 4: Table S3. — Analysis of CH50 values in CSF from various prion diseases according to the intervals from disease onset to sampling. (DOCX 20 kb) [file 40249_2016_128_MOESM4_ESM.docx]

Supplemental Table 3. Analysis of CH50 values in CSF from various prion diseases according to the intervals from disease onset to sampling

| Intervals  (months) | sCJD | gCJD | FFI | Non-CJD |
| --- | --- | --- | --- | --- |
| 0-3 | 100.541 (4.557, 430.887) | 87.773 (22.392, 109.236) | 94.716 | 174.471 (18.051, 478.771) |
| 4-6 | 130.375 (33.888, 474.353) | 83.272 (70.038, 99.244) | 84.332 (21.427, 98.338) | 129.489 (12.407, 435.22) |
| 7-9 | 140.452 (55.489, 242.435) | 84.634 (77.51, 84.634) | 88.166 (79.201, 105.341) | 93.851 (14.719, 329.599) |
| 〉9 | 96.643 (75.947, 176.363) | 78.894 | 94.927 (41.892, 109.748) | 164.802(14.824, 365.022) |
| p value^a^ | 0.615 | 0.935 | 0.727 | 0.479 |

^a^ Kruskal-Wallis H test
