# Supplementary figures and images for: Low activity of complement in the cerebrospinal fluid of the patients with various prion diseases
Source: Infect Dis Poverty. 2016 May 3;5:35. doi: 10.1186/s40249-016-0128-7 (PMC4853859; doi:10.1186/s40249-016-0128-7)

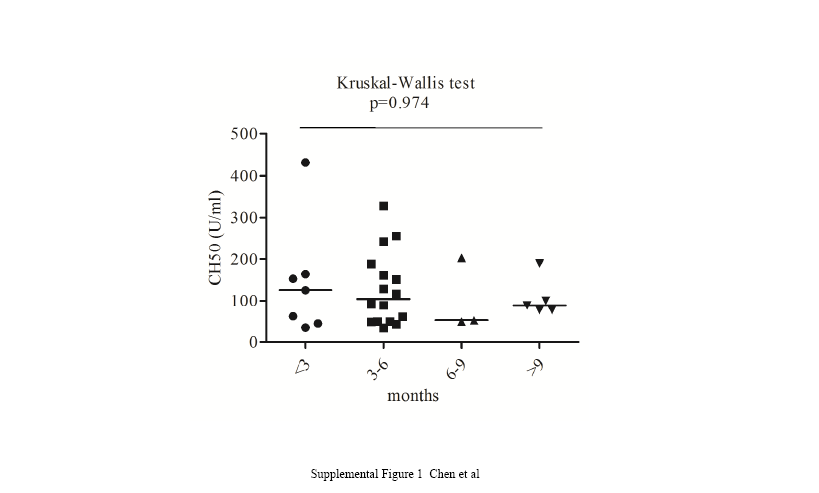

Supplement: Additional file 5: Figure S1. — Description of CH50 values present within CSF from sampling until respective subject’s death, derived from 31 sCJD cases. X-axis represents the interval time and Y-axis represents the CH50 values present within the CSF. P values among the groups are indicated above. (TIF 46 kb) [file 40249_2016_128_MOESM5_ESM.tif]
